# Machine Learning Predicts Phenoconversion from Polysomnography in Isolated REM Sleep Behavior Disorder

**DOI:** 10.3390/brainsci14090871

**Published:** 2024-08-28

**Authors:** Matteo Cesari, Andrea Portscher, Ambra Stefani, Raphael Angerbauer, Abubaker Ibrahim, Elisabeth Brandauer, Simon Feuerstein, Kristin Egger, Birgit Högl, Antonio Rodriguez-Sanchez

**Affiliations:** 1 Department of Neurology, Medical University of Innsbruck, 6020 Innsbruck, Austria; 2 Department of Computer Science, University of Innsbruck, 6020 Innsbruck, Austria

**Keywords:** alpha-synucleinopathy, biomarker, iRBD, phenoconversion, PSG, REM sleep

## Abstract

Isolated rapid eye movement (REM) sleep behavior disorder (iRBD) is a prodromal stage of alpha-synucleinopathies. This study aimed at developing a fully-automated machine learning framework for the prediction of phenoconversion in patients with iRBD by using data recorded during polysomnography (PSG). A total of 66 patients with iRBD were included, of whom 18 converted to an overt alpha-synucleinopathy within 2.7 ± 1.0 years. For each patient, a baseline PSG was available. Sleep stages were scored automatically, and time and frequency domain features were derived from electromyography (EMG) and electroencephalography (EEG) signals in REM and non-REM sleep. Random survival forest was employed to predict the time to phenoconversion, using a four-fold cross-validation scheme and by testing several combinations of features. The best test performances were obtained when considering EEG features in REM sleep only (Harrel’s C-index: 0.723 ± 0.113; Uno’s C-index: 0.741 ± 0.11; integrated Brier score: 0.174 ± 0.06). Features describing EEG slowing had high importance for the machine learning model. This is the first study employing machine learning applied to PSG to predict phenoconversion in patients with iRBD. If confirmed in larger cohorts, these findings might contribute to improving the design of clinical trials for neuroprotective treatments.

## 1. Introduction

Parkinson’s disease (PD), dementia with Lewy bodies (DLB), and multiple-system atrophy (MSA) are neurodegenerative disorders classified as alpha-synucleinopathies, which are characterized by the aggregation of misfolded alpha-synuclein protein [1]. Despite advancements in the understanding of pathophysiological mechanisms underlying alpha-synucleinopathies, these are still unclear, and currently no treatment is available to alter the disease progression. Recent scientific endeavors have focused on the identification of alpha-synucleinopathies in their prodromal stage [2]. This critical period provides a window of opportunity for early detection and intervention, in a phase when treatments may significantly modify the disease evolution [3].

Rapid eye movement (REM) sleep behavior disorder (RBD) is characterized by abnormal muscle activity and vivid, intense, and often violent dream enactment during REM sleep [4]. In healthy individuals, REM sleep entails a suppression of muscle activity (termed muscle atonia), preventing the physical enactment of dream content. Patients with RBD experience a condition called REM sleep without atonia (RWA), associated with a spectrum of behaviors ranging from jerks to more elaborate actions like kicking, punching, or complex movements. According to the American Academy of Sleep Medicine (AASM) [4] and recent guidelines by the International RBD Study Group [5], the diagnosis of RBD necessitates a full-night (video) polysomnography (v-PSG). A v-PSG consists of the simultaneous recording of various physiological indicators, including electroencephalography (EEG), eye movements, muscular activity recorded with electromyography (EMG), cardiorespiratory activity, and simultaneous video recording, resulting in a substantial amount of electrophysiological data.

A large body of research evidence has shown that RBD in its isolated form (iRBD, i.e., without other associated overt neurological disease) represents the prodromal stage of alpha-synucleinopathies [6]. Various long-term follow-up studies have confirmed iRBD to be a prodromal stage of alpha-synucleinopathies, as it has been shown that the risk for developing neurodegenerative disease is 33.5% at a five-year follow-up, 82.4% at 10.5 years, and 96.6% at 14 years, with over 43% of those patients developing PD and 25% developing DLB [7]. Patients with iRBD represent, therefore, an ideal cohort to assess neuroprotective and disease-modifying treatments [8].

Due to the variable time between iRBD diagnosis and phenoconversion, there is a need to identify biomarkers of phenoconversion that could track the progression of neurodegeneration. In recent years, various biomarkers indicating the risk for phenoconversion have been investigated in patients with iRBD [6,8,9]. These include brain imaging, motor function, cognition, vision, olfaction, and autonomic function, along with tissue, genetic, and bio-fluid biomarkers [6]. However, none of these are universally accepted as the best biomarker to be used in the context of clinical trials [8].

Given that the diagnosis of RBD requires v-PSG, researchers have explored electrophysiological biomarkers from PSG that can track neurodegeneration and predict phenoconversion in patients with iRBD [6,10]. Some works showed that specific biomarkers (e.g., sleep spindles [11], sleep-related eye movements [12], and sleep structure [13]) are abnormal in iRBD and even more altered in patients with overt alpha-synucleinopathies, thus suggesting the potential of these biomarkers to track the evolution of neurodegeneration. Other works have shown that patients with iRBD phenoconverting to an overt alpha-synucleinopathy had increased amounts of RWA [14,15,16,17], specific sleep EEG [18,19,20], and resting-state EEG features [21,22,23,24,25] at baseline compared to patients who remained isolated. While machine learning algorithms have been employed in several works to discriminate patients with iRBD by using PSG data [26,27,28,29], to the best of the authors’ knowledge, only two works employed machine learning to predict phenoconversion [22,24]. Both works employed resting EEG data for the prediction [22,24]. One study described phenoconversion dichotically, without considering the time between baseline resting-state EEG and conversion [22], while the other included the time to phenoconversion by employing a survival machine learning model [24]. No study investigated whether it is possible to predict phenoconversion in patients with iRBD using data recorded during v-PSG and machine learning. As PSG is necessary for the diagnosis of iRBD, having a model that could instantly predict the time to phenoconversion to an overt alpha-synucleinopathy would be possibly helpful when neuro-protective and disease-modifying treatments will be available.

In this work, we hypothesize that it is possible to predict the conversion from iRBD to overt alpha-synucleinopathies by extracting and selecting features from data recorded in a baseline full-night v-PSG. Specifically, our aim lies in the utilization of features derived from EEG and EMG signals to train and test supervised survival machine learning models for prediction of phenoconversion in patients with iRBD. The main contributions of this work are as follows: (i) the training and testing of a fully automatic algorithm for the prediction of phenoconversion in patients with iRBD by employing data recorded in a baseline v-PSG and (ii) the analysis of sleep EEG and EMG features that are the most important ones for the prediction of phenoconversion.

## 2. Materials and Methods

### 2.1. Patients and Recordings

This study utilized baseline v-PSG data of a cohort of 66 patients with iRBD conducted at the Sleep Disorders Clinic, Department of Neurology, Medical University of Innsbruck, Austria between 2004 and 2020. One baseline v-PSG was available for each subject. V-PSGs were carried out in accordance with international guidelines adopted at the time of the recordings [30,31,32]. This retrospective study was approved by the ethical committee of the Medical University of Innsbruck (study 1054/2020).

Each v-PSG consisted of the recording of at least the following signals: four EEG signals (C3-M2, C4-M1, O1-M2, and O2-M1), vertical and horizontal electrooculogram, chin and bilateral tibialis anterior (TA) EMG, electrocardiogram, oxygen saturation, and signals to monitor respiration (abdominal and thoracic movements, tracheal vibrations, and nasal-pneumo airflow). The EEG signals were sampled at a frequency of 250 Hz. The EMG signals were sampled either at a frequency of 500 Hz (20 subjects) or 1 kHz (46 subjects). Sleep stages were manually scored in 30 s epochs as either wakefulness, REM sleep, or one of the three non-REM sleep stages (N1, N2, N3 sleep) by experienced technicians. Similarly, the technicians also manually scored respiratory events. Manual scoring was performed according to international rules adopted at the time of the recordings [30,31,32]. Periodic leg movements were scored with a validated automatic algorithm [33].

All patients were diagnosed with iRBD according to the applicable international criteria at the time of the recordings [34,35,36] and fulfilled recently proposed criteria [5]. At baseline, no patient had overt alpha-synucleinopathy. After an average of 4.5 years (standard deviation: 3.1 years), all patients underwent a neurological follow-up evaluation. Classification into distinct groups was based on their respective diagnoses, yielding the following categories: 48 patients (72.7%) were still diagnosed with iRBD, while 18 patients (27.3%) phenoconverted within 2.7 ± 1.0 years (range 1.0–4.3 years) to an overt alpha-synucleinopathy (13 of them to PD, 4 to DLB, and 1 to MSA). The two groups did not differ in demographics, medication intake, sleep macro-structure, or sleep comorbidities at the baseline v-PSG, as shown in Table 1. In this study, only data from the baseline v-PSG were included in the analyses.

### 2.2. Automatic Sleep Stage Scoring

To fulfill the objective of developing a fully automated algorithm, and to overcome inter-rater variability between manual scorers [37], the validated YASA algorithm [38] was applied to score sleep stages in 30 s epochs (Python package yasa:0.6.3). Briefly, for each PSG, we extracted the raw C4-M1 EEG signal and gave it in input to the YASA algorithm, which scored each 30 s epoch as either wakefulness, N1, N2, N3, or REM sleep. To assess the reliability of the automatically assigned sleep stages, the overall and sleep stage-specific Cohen’s Kappa coefficient (κ) between the automatically and manually scored sleep stages were computed. Manual and automatic scorings were time-synchronized and aligned to the raw data.

### 2.3. Feature Extraction

**Figure 1:**
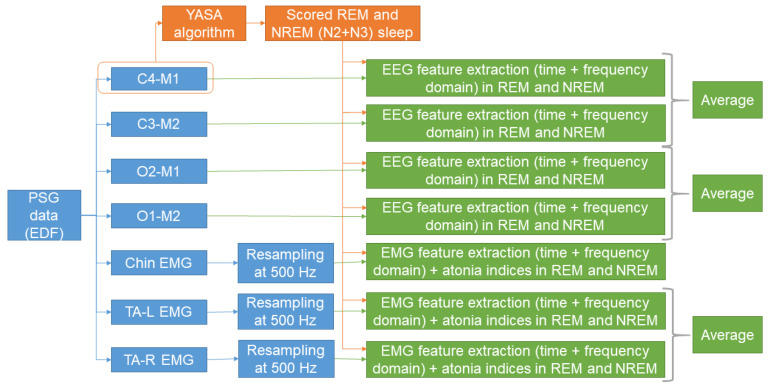
Schematic representation of the process of feature extraction. Based on the automatically scored sleep stages, features were derived from central and occipital EEG, and from chin and TA EMG channels. A detailed description is reported in Section 2.2 and Section 2.3, and the relative subsections.

To build a machine learning survival model for predicting phenoconversion in patients with iRBD, we extracted several features from each patient. The features were extracted from EEG and EMG channels. This choice was based on previous studies reporting that sleep EEG and EMG might hold important information with regards to the phenoconversion [14,15,16,17,18,19,20]. Feature extraction was performed with scripts written in Matlab R2021b (MathWorks^®^, Natick, MA, USA). Figure 1 shows a schematic representation of the process of feature extraction.

#### 2.3.1. EEG Features

The C3-M2, C4-M1, O1-M2, and O2-M1 EEG signals were notch-filtered (50 Hz) to remove electricity noise, and bandpass-filtered between 0.3 and 35 Hz, as recommended by international criteria [39]. After this preprocessing, each EEG signal was divided into non-overlapping 5 s windows. With reference to a previous work [27], and based on previous studies showing that EEG slowing might be a possible marker of phenoconversion [19,20], several features were derived from each window, both in time domain and frequency domain. To derive frequency domain features, the Welch method (1 s windows with 50% overlap, resolution of 0.5 Hz) was employed to calculate the power spectrum. The average of the features across the 5 s windows was calculated for each patient both in automatically scored REM and in NREM sleep (defined as N2 and N3 sleep). Finally, the values of the two central and two occipital derivations were also averaged for each patient. Table 2 reports a detailed description of the features extracted from the EEG signals.

#### 2.3.2. EMG Features

The following EMG channels were considered for feature extraction: chin EMG, TA left EMG, and TA right EMG. All signals were analyzed at a sampling frequency of 500 Hz, thus undersampling the ones recorded at 1 kHz. Resampling was performed with the Matlab function *resample*, that applies a finite impulse response antialiasing lowpass filter to prevent signal distortion. Following international criteria [39], EMG signals were notch-filtered at 50 Hz and bandpass-filtered between 10 and 100 Hz. As for the EEG signals, EMG signals were divided into non-overlapping 5 s windows, and from each window, several time domain and frequency domain features were calculated. The features were calculated based on a previous work [27]. To calculate the frequency domain features, the periodogram method was employed for calculation of the power spectrum. The average of the features across the 5 s windows was calculated for each patient both in automatically scored REM and in NREM sleep (defined as N2 and N3 sleep). Finally, the features derived for the two TA EMG channels were averaged. Table 3 reports a detailed description of the features extracted from the EMG signals.

In addition to these features, based on the previous research suggesting that increased muscular tone is associated to phenoconversion [14,15,16,17,18,19,20], we calculated the REM and NREM atonia index in the chin and the TA channels, following the original description of the algorithm [45,46]. As for the other features, automatically scored REM and NREM (defined as N2 and N3 sleep) were considered to compute the REM and NREM atonia indices.

### 2.4. Machine Learning Model Training and Testing

**Figure 2:**
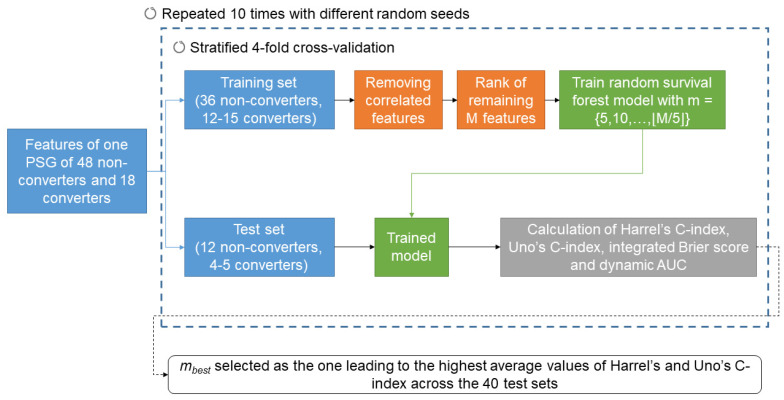
Schematic representation of the process of machine learning model training and testing. A detailed description is reported in Section 2.4.

In order to predict phenoconversion, we employed a machine learning approach based on survival analysis [24]. As previously performed [24], we adopted a machine learning survival approach and not a conventional dichotomous classifier, as machine learning survival models allow to predict when and how fast the phenoconversion occurs. A schematic representation of the process of machine learning model training and testing is shown in Figure 2.

The dataset was divided into 4 subject-based stratified folds and, in a 4-fold cross-validation approach, we used 3 folds as training set and 1 as testing set. A cross-validation approach was preferred to a hold-out one due to the small sample size [47]. Having 4 folds allowed having 4 or 5 converters and 12 non-converters in each fold. For each training set, highly correlated features (Spearman correlation coefficient > 0.8) were removed. The remaining *M* features were then ranked, based on the absolute value of coefficients obtained with multivariate Cox proportional hazard regressions on each training set (highest rank given to the feature with the highest absolute coefficient). A random survival forest (100 estimators, minimum samples split = 6 and minimum samples leaf = 3) was then trained on each training set by considering the *m* features with the highest rank, with $m\in \left\{5,\;10,\ldots ,\left\lfloor M/5\right\rfloor \right\}.$ The trained model was then tested on each respective test fold. The performance of the prediction was estimated with Harrel’s C-index, Uno’s C-index, integrated Brier score, and the average time-dependent area under the receiver operating characteristic curve (AUC, calculated for times = 2, 4, …, 10 years). The C-index is a measure of the ability of the model to correctly rank the survival time of the patients and it has been shown that Uno’s C-index is more accurate than Harrel’s C-index for imbalanced problems [48]. The higher the C-index, the better the survival time ranking is performed. The integrated Brier score indicates the overall accuracy of the model, considering both predicted survival times and the observed survival times. The lower the integrated Brier score, the more accurate the model is. Finally, the dynamic AUC is a measure of how the model discriminates between converters and non-converters at different time points. Permutation feature importance was employed to represent the importance of each variable in the model.

Ten runs of the four-fold cross-validation were repeated with different random seeds, thus 40 models were trained and tested, when considering *m* features. The best number of features (*m_best_*) was identified in correspondence to the highest average values of Harrel’s and Uno’s C-index across the 40 test sets.

Several experiments were conducted by considering the following set of features: (i) EMG features calculated in REM only (EMG_REM_, 46 features); (ii) EMG features calculated in NREM sleep only (EMG_NREM_, 46 features); (iii) EEG features calculated in REM sleep only (EEG_REM_, 52 features); (iv) EEG features calculated in NREM sleep only (EEG_NREM_, 52 features); (v) the combination of EMG features calculated in REM and NREM sleep (EMG_REM_ + EMG_NREM_, 92 features); (vi) the combination of EEG features calculated in REM and NREM sleep (EEG_REM_ + EEG_NREM_, 104 features); (vii) the combination of EMG and EEG features calculated in REM sleep (EMG_REM_ + EEG_REM_, 98 features); (viii) the combination of EMG and EEG features calculated in NREM sleep (EMG_NREM_ + EEG_NREM_, 98 features); and (ix) the combination of EMG and EEG features calculated in REM and NREM sleep (196 features).

Machine learning model training and testing were conducted in Python 3.9.19, with the following packages: scikit-survival 0.22.2, feature-engine 1.6.2, numpy 1.26.4, scikit-learn 1.3.2, pandas 2.2.2, and matplotlib 3.8.4.

### 2.5. Code

The code for feature extraction and machine learning training and validation is available at https://github.com/macesari/predictRBD (accessed on 21 July 2024).

## 3. Results

The YASA algorithm for sleep stage scoring had an overall agreement with manual sleep stage scoring of κ = 0.56 ± 0.14, which is in line with the agreement between manual scorers [37]. Table 4 shows the detailed results per sleep stage.

Table 5 reports, for each experiment, the best number of features (*m_best_*), and the average and standard deviation of Harrel’s C-index, Uno’s C-index, and time-dependent AUC across the 40 test sets. The results indicate that the best prediction performance was achieved when considering only EEG features calculated in REM sleep and only five features. In this case, phenoconversion could be predicted with Harrel’s C-index of 0.723 ± 0.113, Uno’s C-index of 0.741 ± 0.11, integrated Brier score of 0.174 ± 0.06, and AUC of 0.780 ± 0.145.

**Figure 3:**
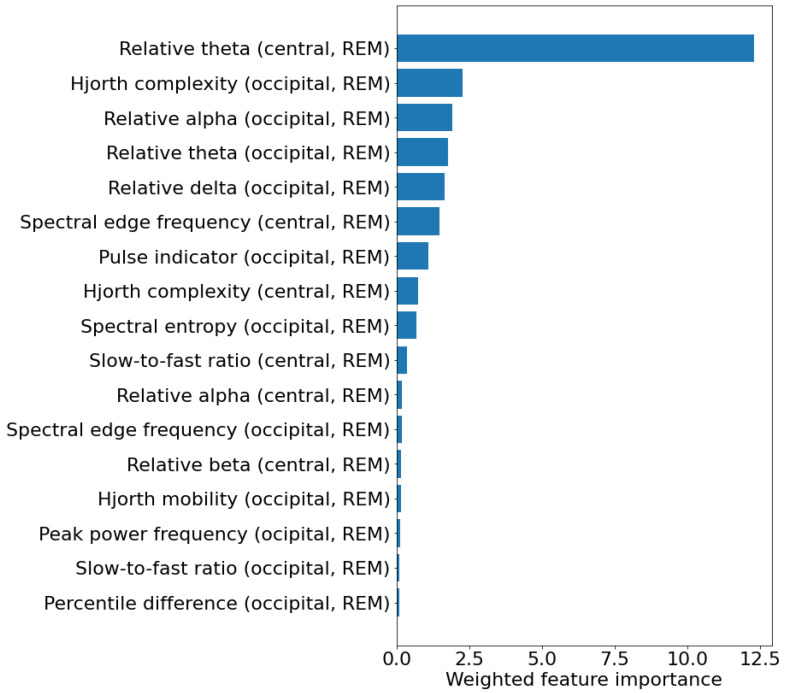
Weighted feature importance across the 40 test sets for the experiment considering 5 EEG features calculated in REM sleep. For each feature, the weighted feature importance was obtained by multiplying the average permutation-based feature importance across the test sets by the percentage of models in which the feature was selected. The unit of measure of the weighted feature importance is arbitrary.

As different features were selected in the different training set, a weighted feature importance score was calculated for each feature. This value was obtained by multiplying the average permutation-based feature importance across the test sets by the percentage of models in which the feature was selected. Figure 3 shows the weighted feature importance for the best model. Among the features, the one showing the highest weighted importance is the relative power in the theta band in the central channels. Table 6 shows the values of the 10 most important features in the two patient groups, and their hazard ratio and relative *p*-value (calculated with univariate Cox proportional hazard regression), indicating how they are associated with phenoconversion. Most of the features are related to the frequency content of the EEG and indicate a general slowing of REM sleep EEG in iRBD patients that phenoconverted. In particular, converters showed increased relative theta power in the central channels (*p* = 0.033), decreased alpha power in the occipital channels (*p* = 0.042), and increased slow-to-fast ratio in the central channels (*p* = 0.008).

## 4. Discussion

To the best of the authors’ knowledge, this is the first study evaluating whether features extracted from EMG and EEG recorded during PSG allow prediction of phenoconversion to an overt alpha-synucleinopathy in patients with iRBD with a machine learning approach. The best results (Harrel’s C-index of 0.723 ± 0.113, Uno’s C-index of 0.741 ± 0.11, integrated Brier score of 0.174 ± 0.06, and AUC of 0.780 ± 0.145) could be achieved by considering only features derived from EEG in automatically scored REM sleep. Analysis of feature importance revealed that features expressing EEG slowing had a high importance in the prediction model.

Previous studies mainly adopted classical statistical approaches analyzing baseline features distinguishing converters from non-converters [14,15,16,17,18,19,20,21,23]. These studies lack a clear separation of data into training and testing sets, potentially leading to limited generalizability. In fact, classical statistical approaches focus on group differences, while machine learning methods focus on finding generalizable predictive patterns [50]. As previously mentioned, only two studies employed machine learning to predict phenoconversion. In one study, the authors dealt with the problem considering it a dichotomous classification problem [22]. In that study, the authors showed that, by using spectrograms derived from the resting state EEG, a convolutional neural network could differentiate with 73% accuracy converters from the pooled group of non-converters and controls. The authors did not attempt the differentiation only between converters and non-converters and, furthermore, did not report performance metrics like precision and recall that would help to better estimate the generalizability of the network. In a more recent study [24], the authors also predicted phenoconversion with a machine learning approach with survival analysis using resting state EEG. In that work, the authors showed that phenoconversion could be predicted with Harrel’s C-index of 0.775 and integrated Brier score of 0.114 on the test data coming from the same clinic of the training data. The authors also evaluated the model on an external dataset, achieving a Harrel’s C-index of 0.561 and an integrated Brier score of 0.128. Our results are similar to the ones obtained in that work, thus supporting the validity of our study.

In our work, we attempted the classification in a fully automated setup, by performing sleep staging with an open-access and previously validated algorithm [38]. When compared with manual scoring, the algorithm has overall agreement in line with previous reports [37]. The overall agreement is in line also with an automatic sleep staging algorithm which has been tested on patients with RBD [27]. Thus, it can be assumed that the employed automatic sleep staging approach is acceptable. As features were calculated in automatically scored REM and NREM sleep, this ensures the reliability of the features themselves. The calculation of Cohen’s Kappa should not be strictly seen as a validation of the YASA algorithm in our population due to the lack of multiple manual sleep stage scoring annotations and because of the adoption of slightly different scoring rules over the years of data collection.

Among the several sets of features tested for prediction of phenoconversion, EEG features derived in REM sleep led to the best performances. The feature analysis revealed that the most important features were the ones describing EEG slowing, expressed in terms of significant increase in theta bandpower, reduction in alpha bandpower, and increase in the slow-to-fast ratio. Our results substantiate a body of the literature showing that EEG slowing is an important feature differentiating converters from non-converters. Interestingly, the pattern of slowing has been reported both in sleep [19,20] and in resting state EEG [21,22,23,24]. The analysis of the most important features also revealed other trends in converters compared with non-converters, including a tendency to increased Hjorth complexity. Recent works showed that patients with overt Parkinson’s disease have increased Hjorth complexity in resting state EEG compared with controls [51,52]. Taken together, the results suggest that increased EEG Hjorth complexity might be a biomarker of synuclein-related neurodegeneration.

While previous works showed that increased RWA is an important biomarker of phenoconversion [14,15,16,17,18,19,20], our analysis showed that the model including only EMG features in REM sleep achieved an average C-index below 0.55 and an average AUC below 0.62. It can be argued that the automatic sleep stage scoring algorithm might have scored epochs with RWA as wakefulness or N1 sleep, therefore missing the increased muscular tone in REM sleep as an important discriminating feature between converters and non-converters. To check for this, by following the same methodology, we trained random survival forest models by considering EMG features calculated in manually scored sleep and we achieved Harrel’s C-index of 0.602 ± 0.113, Uno’s C-index of 0.611 ± 0.107, integrated Brier score of 0.200 ± 0.052, and AUC of 0.699 ± 0.124. These performance metrics are still lower than the ones we obtained by considering EEG features in automatically scored REM sleep, thus indicating that EEG changes might be more informative than increased muscular tone in REM sleep for predicting phenoconversion in patients with iRBD. These findings are in line with a recent deep learning work, which showed that EEG is superior to EMG for the identification of patients with iRBD [28].

This work confirms the growing importance of machine learning for analysis of PSG in patients with RBD. Some previous work showed how both classical machine learning and deep neural networks can be successfully employed for (semi)-automatic identification of patients with iRBD [26,27,28,29]. This work adds to the literature, showing that machine learning applied to PSG data can be helpful to predict phenoconversion.

The main strength of the study is that it is the first one employing machine learning to sleep data to predict phenoconversion in patients with iRBD. Limitations of the study include, first of all, its retrospective design, the inclusion of data from only one center, and the lack of an external set for validation of the models. Due to the small sample size, the test sets included only 4 or 5 converters and 12 non-converters. This explains the high standard deviation in the performances. As a consequence of the limited amount of data, this study may be susceptible to bias and reduced generalizability. To increase the number of data points in the positive class, we aggregated subjects who converted to different alpha-synucleinopathies, including Parkinson’s disease, dementia with Lewy bodies, and multiple system atrophy. However, this approach might have introduced some level of noise into the findings due to the diversity of conditions included. Concerning the methodology, our approach has the strength that it was carefully designed to avoid any type of overfitting and consisted of a feature selection for each training set and the subsequent training of a random survival forest model to predict phenoconversion. We reported the average performance across all folds and runs, ensuring a robust evaluation of the model while avoiding circular analysis. A limitation in our methodological approach was the lack of a systematic hyperparameter selection. In fact, we used the default hyperparameters of random survival forest and we did not systematically optimize them with a grid search or random search approach due to the small sample size. It cannot be excluded that other parameters or other survival machine learning algorithms could have led to better results. Furthermore, based on previous works [53,54], we selected to extract features on 5 s windows, but we cannot exclude that other window lengths would have led to improved results. Finally, we did not test the effect of using other thresholds for identifying correlated features in the feature selection process.

Future works should include a larger multi-centric cohort to investigate in generalizability of the findings, and implement systematic hyperparameter tuning to potentially improve model performance. The availability of a large multi-centric cohort will also allow to externally validate the robustness of the algorithm to any population bias. Furthermore, future studies should compare models using only PSG data to models using other modalities, e.g., resting state EEG or imaging, or a combination of them. Finally, longitudinal studies will be needed to assess the long-term accuracy of the predictive models and their clinical utility.

## 5. Conclusions

This study showed that EEG features derived from automatically scored REM sleep allowed to predict phenoconversion in patients with iRBD with Harrel’s C-index of 0.723 ± 0.113, Uno’s C-index of 0.741 ± 0.11, integrated Brier score of 0.174 ± 0.06, and AUC of 0.780 ± 0.145 by employing random survival forest models. The analysis of relevant features showed that converters are characterized by EEG slowing. This study, therefore, shows the potential of PSG biomarkers for the prediction of phenoconversion. If confirmed in larger longitudinal and multi-centric studies, future clinical trials might consider these PSG biomarkers and machine learning survival models to identify patients with iRBD with a higher likelihood to phenoconvert in the short term.

## Figures and Tables

**Table 1:** Demographics, sleep structure and related events, sleep comorbidities, and medication intake at baseline in converters and non-converters. Normality distribution for each variable was checked with Shapiro–Wilk tests. In case of normally distributed variables, values are shown as mean ± standard deviation and the groups were compared with Student *t*-tests. Otherwise, values are shown as median and inter-quartile ranges, and the groups were compared with Mann–Whitney tests. Categorical variables were compared with Chi-squared tests.

Parameter	Non-Converters	Converters	*p*-Value
Number	48	18	-
Males (%)	95.8	83.3	0.087
Age (years)	68 [63–73]	72.5 [66–76]	0.160
Time in bed (min)	485 [473–492.5]	485.5 [476–509]	0.324
Total sleep time (min)	382.5 ± 57.8	399.9 ± 77.8	0.327
Sleep period time (min)	463.5 [443.5–472.5]	467 [415–478]	0.560
Sleep efficiency (%)	80.45 [69.45–88.4]	84.95 [79.3–87.1]	0.476
Sleep latency (min)	14 [8.55–25.3]	12.5 [4.3–23.8]	0.293
REM latency (min)	97.5 [64.5–191.75]	97.5 [75–121.5]	0.703
Wake (%SPT)	13.7 [8.7–21.7]	13.2 [10.0–15.77]	0.757
N1 (%SPT)	12.5 [3.1–19.6]	14.0 [9.6–18.8]	0.658
N2 (%SPT)	47.4 [37.2–56.2]	44.9 [39.6–54.7]	0.713
N3 (%SPT)	2.3 [0.0–8.2]	5.6 [0.0–8.9]	0.667
REM (%SPT)	14.8 ± 7.4	16.2 ± 7.9	0.523
AHI (/h)	2.9 [1.5–5.3]	2.9 [0.3–8.8]	0.857
AHI in REM (/h)	2.5 [0.0–8.1]	2.1 [0.0–6.5]	0.724
PLMS index (/h)	20.3 [6.7–57.6]	24.0 [11.4–47.0]	0.812
Sleep-related breathing disorder (%)	89.6	77.8	0.213
Restless legs syndrome (%)	22.9	27.8	0.681
Antidepressants (%)	43.8	22.2	0.108
Benzodiazepines (%)	8.3	16.7	0.327
Antipsychotics (%)	14.6	5.6	0.316
Beta-blockers (%)	14.6	5.6	0.316
Antiepileptics (%)	10.4	11.1	0.934
Dopamine agonists (%)	2.1	5.6	0.463
Clonazepam (%)	6.3	16.7	0.189

AHI—apnea-hypopnea index; PLMS—periodic limb movement during sleep; REM—rapid eye movement; SPT—sleep period time.

**Table 2:** Features extracted from each 5 s window of each EEG signal.

Feature Name	Type	Description
Zero-crossing rate	Time domain	The number of zero-crossings, normalized by the window length
Hjorth parameters	Time domain	The three Hjorth parameters (activity, mobility, and complexity) [40]
Time domain properties	Time domain	Time domain properties derived using the log-power and the log-amplitude of each derivative, up to the 10th derivative [41]
Percent differential	Time domain	Difference between the 75th and 25th percentile of signal amplitude
Coastline	Time domain	The sum of the rectified sample derivative of the signal
Root mean square	Time domain	The root mean square of the signal
Variance	Time domain	The variance of the signal
Peak-to-peak	Time domain	The difference between the maximum and minimum peak of the signal
Crest factor	Time domain	The ratio between the absolute peak of the signal and the root mean square
Form factor	Time domain	The ratio between the root mean square and the average of the rectified signal
Pulse indicator	Time domain	The ratio between the absolute peak of the signal and the average of the rectified signal
Teager–Kaiser energy operator	Time domain	The energy of the signal, calculated according to [42]
Permutation entropy	Time domain	A non-linear measure that characterizes the complexity of the signal, calculated to the 10th order [43]
Shannon entropy	Time domain	The normalized Shannon entropy [44], calculated with the number of bins equal to the square root of the signal samples
Peak-power frequency	Frequency domain	The frequency at which the maximum in the power spectrum is achieved
Spectral edge frequency	Frequency domain	The frequency below which 95% of the signal power is contained
Relative power spectra	Frequency domain	Relative power calculated in the delta (0.5–4 Hz), theta (4–8 Hz), alpha (8–13 Hz), and beta (13–35 Hz) bands.
Slow-to-fast ratio	Frequency domain	The ratio between the relative powers in delta and theta bands with respect to the relative powers in alpha and beta bands

**Table 3:** Features extracted from each 5 s window of each EMG signal.

Feature Name	Type	Description
Zero-crossing rate	Time domain	The number of zero-crossings, normalized by the window length
Root mean square	Time domain	The root mean square of the signal
Variance	Time domain	The variance of the signal
Peak-to-peak	Time domain	The difference between the maximum and minimum peak of the signal
Crest factor	Time domain	The ratio between the absolute peak of the signal and the root mean square
Form factor	Time domain	The ratio between the root mean square and the average of the rectified signal
Pulse indicator	Time domain	The ratio between the absolute peak of the signal and the average of the rectified signal
Teager–Kaiser energy operator	Time domain	The energy of the signal, calculated according to [42]
Permutation entropy	Time domain	A non-linear measure that characterizes the complexity of the signal, calculated to the 10th order [43]
Shannon entropy	Time domain	The normalized Shannon entropy [44], calculated with the number of bins equal to the square root of the signal samples
Shannon entropy of rectified signal	Time domain	The normalized Shannon entropy of the rectified signal [44], calculated with the number of bins equal to the square root of the signal samples
Normalized Wilson amplitude	Time domain	The number of samples with absolute sample derivative over three times a threshold ^1^, normalized by the signal length
Myopulse indicator	Time domain	The percentage of samples with amplitude over three times a threshold ^1^
Normalized integral	Time domain	The sum of the rectified signal, normalized by its length
Normalized wavelength	Time domain	The sum of the rectified sample derivative of the signal, normalized by its length
Energy	Time domain	The sum of the signal samples squared
75th percentile	Time domain	The 75th percentile of the rectified signal
Fractal exponent	Frequency domain	The negative slope of the spectral density using a logarithmic on both the frequency and power.
Gamma power	Frequency domain	The absolute power in the frequency 30–45 Hz
Peak-power frequency	Frequency domain	The frequency at which the maximum in the power spectrum is achieved
Spectral entropy	Frequency domain	A measure of the random process uncertainty from the frequency distribution.
Spectral edge frequency	Frequency domain	The frequency below which 95% of the signal power is contained

^1^ Threshold defined as three times the standard deviation of the signal in REM or NREM sleep.

**Table 4:** Cohen’s Kappa coefficient measuring the agreement for automatically and manually scored sleep stages. The table shows the mean ± standard deviation across the subjects.

	All Patients	Non-Converters	Converters
κ (overall)	0.56 ± 0.14	0.55 ± 0.13	0.52 ± 0.08
κ (W)	0.61 ± 0.17	0.61 ± 0.18	0.61 ± 0.14
κ (N1)	0.17 ± 0.11	0.18 ± 0.11	0.17 ± 0.10
κ (N2)	0.60 ± 0.15	0.61 ± 0.16	0.58 ± 0.12
κ (N3)	0.53 ± 0.25	0.54 ± 0.24	0.48 ± 0.26
κ (REM)	0.56 ± 0.26	0.57 ± 0.26	0.52 ± 0.26

**Table 5:** Performance for the prediction of phenoconversion across the test sets. For each experiment, the best number of features (*m_best_*) is shown, together with the Harrel’s C-index, Uno’s C-index, integrated Brier score, and area under the receiver operating characteristic curve (AUC). The values are shown as mean and standard deviation across the 40 test sets, obtained with 10 runs of 4-fold cross-validation.

Experiment	*m_best_*	Harrel’s C-Index	Uno’s C-Index	Integrated Brier Score	AUC
EMG_REM_	15	0.539 ± 0.135	0.548 ± 0.125	0.226 ± 0.07	0.619 ± 0.160
EMG_NREM_	15	0.542 ± 0.124	0.538 ± 0.125	0.243 ± 0.082	0.560 ± 0.165
EEG_REM_	5	0.723 ± 0.113	0.741 ± 0.110	0.174 ± 0.06	0.780 ± 0.145
EEG_NREM_	10	0.558 ± 0.109	0.559 ± 0.118	0.24 ± 0.085	0.602 ± 0.162
EMG_REM_ + EMG_NREM_	20	0.545 ± 0.143	0.553 ± 0.141	0.24 ± 0.087	0.584 ± 0.157
EEG_REM_ + EEG_NREM_	15	0.700 ± 0.139	0.710 ± 0.132	0.194 ± 0.061	0.746 ± 0.152
EMG_REM_ + EEG_REM_	25	0.649 ± 0.145	0.653 ± 0.13	0.214 ± 0.081	0.701 ± 0.178
EMG_NREM_ + EEG_NREM_	25	0.601 ± 0.111	0.594 ± 0.121	0.233 ± 0.084	0.616 ± 0.161
All	50	0.634 ± 0.141	0.639 ± 0.140	0.171± 0.088	0.688 ± 0.171

**Table 6:** Univariate Cox regression analysis of the 10 most important features for the experiment considering 5 EEG features calculated in REM sleep. For each feature, the values are shown in both converters and non-converters as mean and standard deviation across the patients. Hazard ratios are shown with their 95% confidence interval, and significant *p*-values (<0.05) are highlighted in bold. For all the presented features, the proportional hazard assumption was checked by statistical analysis of the scaled Schoenfeld residuals [49]. All features except “Spectral edge frequency (central, REM)” met this assumption.

Feature	Non-Converters (N = 48)	Converters (N = 18)	Hazard Ratio	*p*-Value
Relative theta (central, REM) [%]	16.6 ± 4.5	18.4 ± 5.4	1.101 [1.007–1.203]	**0.033**
Hjorth complexity (occipital, REM) [u.a.]	2.59 ± 0.39	2.73 ± 0.77	1.845 [0.834–4.079]	0.131
Relative alpha (occipital, REM) [%]	13.87 ± 4.07	11.70 ± 4.04	0.878 [0.774–0.995]	**0.042**
Relative theta (occipital, REM) [%]	18.90 ± 4.20	20.39 ± 5.84	1.086 [0.990–1.192]	0.081
Relative delta (occipital, REM) [%]	39.94 ± 6.44	40.00 ± 11.90	1.009 [0.952–1.070]	0.756
Spectral edge frequency (central, REM) [Hz]	18.12 ± 2.30	17.91 ± 3.79	0.890 [0.723–1.094]	0.268
Pulse indicator (occipital, REM) [-]	4.87 ± 0.70	5.10 ± 1.06	1.262 [0.711–2.242]	0.427
Hjorth complexity (central, REM) [u.a.]	2.59 ± 0.39	2.73 ± 0.77	1.845 [0.834–4.079]	0.131
Spectral entropy (occipital, REM) [u.a.]	2.64 ± 0.163	2.64 ± 0.292	0.522 [0.048–5.630]	0.592
Slow-to-fast ratio (central, REM) [-]	3.81 ± 1.36	4.59 ± 3.26	1.307 [1.073–1.590]	**0.008**

u.a.—unit arbitrary.

## Data Availability

The data presented in this study are available on request from the corresponding author (the data are not publicly available due to ethical restrictions).

## Abbreviations

AHI	apnea-hypopnea index
AUC	area under the receiver operating characteristic curve
DLB	dementia with Lewy bodies
EEG	electroencephalography
EEG_REM_	EEG features calculated in REM sleep
EEG_NREM_	EEG features calculated in NREM sleep
EMG	electromyography
EMG_REM_	EMG features calculated in REM sleep
EMG_NREM_	EMG features calculated in NREM sleep
κ	Cohen’s kappa
iRBD	isolated REM sleep behavior disorder
*m_best_*	best number of features
MSA	multiple system atrophy
N1	NREM stage 1
N2	NREM stage 2
N3	NREM stage 3
NREM	non-REM
REM	rapid eye movement
RWA	REM sleep without atonia
PD	Parkinson’s disease
PLMS	periodic leg movements in sleep
PSG	polysomnography
TA	tibialis anterior
v-PSG	video-PSG
W	wakefulness
